# Crossing Boundaries: A Rare Case of Cardiac Dysfunction

**DOI:** 10.7759/cureus.7998

**Published:** 2020-05-06

**Authors:** Laith Ali, Amre Ghazzal, Tariq Sallam, Raja Zaghlol, Brian Cuneo

**Affiliations:** 1 Internal Medicine, MedStar Washington Hospital Center, Washington, DC, USA; 2 Critical Care/Pulmonary Medicine, MedStar Washington Hospital Center, Washington, DC, USA

**Keywords:** cardiac neoplasm, obstructive shock, hepatocellular carcin, transthoracic echocardiogram

## Abstract

Cardiac tumors are relatively rare. Secondary cardiac tumors are by far more common than primary cardiac tumors. Cardiac involvement may occur secondary to hematogenous metastases, direct invasion, or tumor growth into the venous system and extension into the right atrium. Patients can present with a spectrum of conditions, including embolization, obstruction of ventricular outflow tracts, direct invasion of myocardium causing impaired myocardial function, invasion of surrounding tissue, interference with valvular function causing valvular regurgitation, or constitutional non-specific signs and symptoms. Imaging modalities are essential for diagnosis. Management is mostly surgical, but can include other medical strategies as well. We present a case of a 65-year-old male with hepatocellular carcinoma with direct invasion to the heart through the venous system.

## Introduction

Cardiac tumors are exceedingly rare [1]. In one autopsy series, the incidence of primary cardiac tumors was less than 1% [2]. Secondary tumors are 20-40 folds more common [2-4]. Prognosis can be grim in advanced disease, and presentation can even include shock status. Point-of-care echocardiography can help differentiating the type of shock, and it is relatively an easy skill to learn. Management is mostly surgical. Hepatocellular carcinoma (HCC) is one of the tumors that might spread to the heart [5]. We present a rare case of HCC spreading contiguously to the heart, partially obstructing the right ventricular outflow tract (RVOT).

## Case presentation

A 65-year-old male with medical history of treated hepatitis C and polysubstance abuse presented with progressive shortness of breath of one-week duration with productive cough. Laboratory workup suggested the presence of a shock status with elevated lactic acid, acute renal failure, and acute liver failure (Table 1).

**Table 1:** Lab results on presentation. a: blood urea nitrogen, b: alanine aminotransferase, c: aspartate aminotransferase, d: international normalized ratio

Test	Result	Normal range
Sodium	133 mmol/L	137-145 mmol/L
Potassium	6.7 mmol/L	3.5-5.1 mmol/L
Chloride	91 mmol/L	98-107 mmol/L
Bicarbonate	9 mmol/L	21-32 mmol/L
BUN^a^	84 mg/dL	9-20 mg/dL
Creatinine	3.71 mg/dL	0.66-1.50 mg/dL
Anion gap (corrected)	33 (35.5) mmol/L	5-15 mmol/L
Glucose	32 mg/dL	65-140 mg/dL
ALT^b^	452 units/L	15-41 units/L
AST^c^	1498 units/L	3-34 units/L
Total bilirubin	6.1 mg/dL	0.2-1.3 mg/dL
Direct bilirubin	3.0 mg/dL	0.00-0.30 mg/dL
Alkaline phosphatase	130 units/L	45-117 units/L
Albumin	3 gm/dL	3.5-5.0 gm/dL
INR^d^	5	0.8-1.2
Lactic acid	More than 15.0 mmol/L	0.7-2.0 mmol/L
Hemoglobin	15.0 gm/dL	12.5-16.5 gm/dL
Leukocytes	28.1 * 10^9 cells/L	4.0-10.8 (*10^9) cells/L
Platelets	73 * 10^9 cells/L	145-400 (*10^9) cells/L

**Figure 1:**
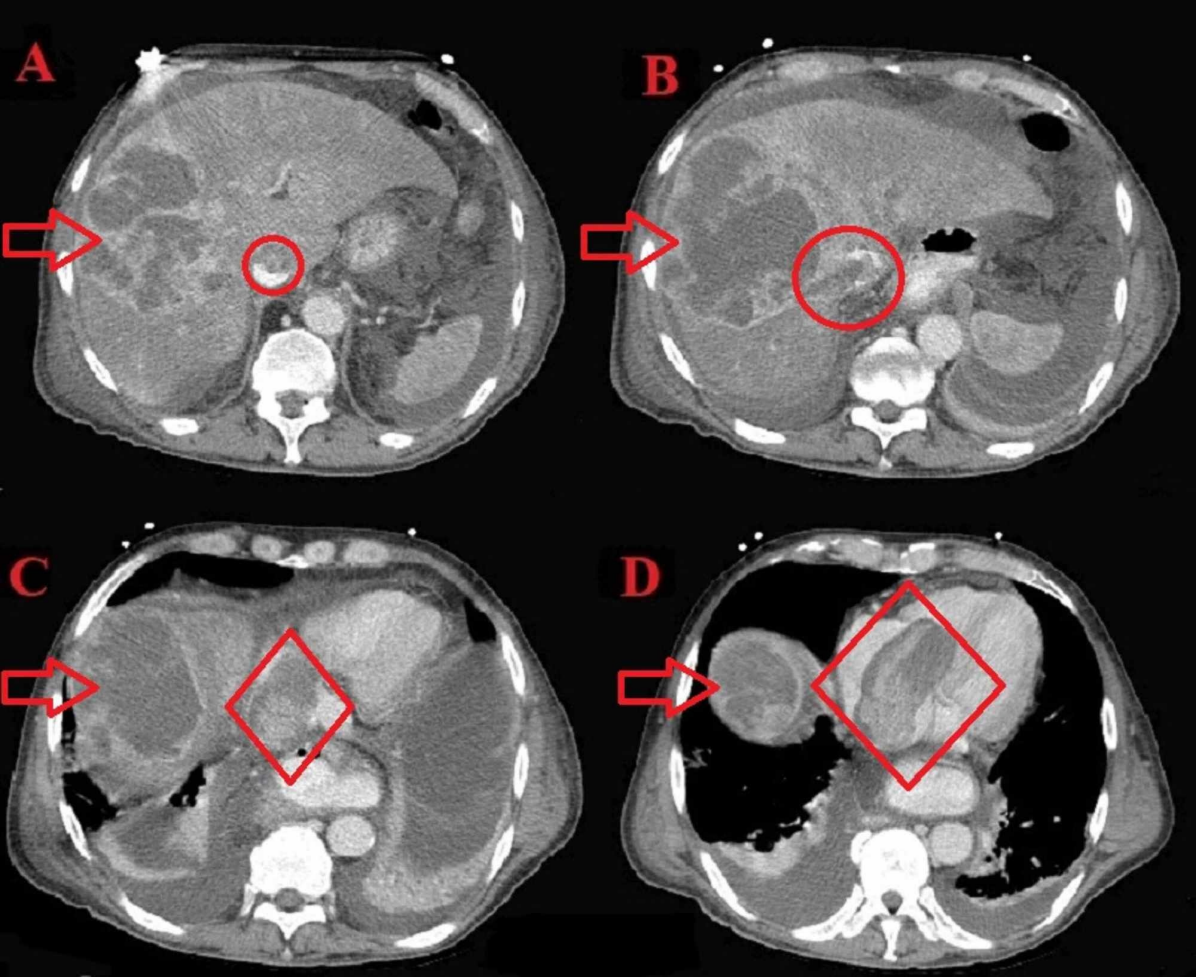
CT scans showing the mass in the liver, right hepatic vein, IVC, RA, and RV. (A) The arrow shows the mass in the liver. The circle shows a filling defect in the IVC suggestive of a mass present in the IVC.(B) The arrow shows the mass in the liver. The circle shows a filling defect extending to the IVC through the right hepatic vein. (C) The arrow shows the mass in the liver. The diamond shows a filling defect in the heart (RA). (D) The arrow shows the mass in the liver. The diamond shows a filling defect in the heart (RV). IVC: inferior vena cava, RA: right atrium, RV: right ventricle

Soon after, he became confused and more hypotensive requiring intubation. Point-of-care echocardiogram was done and showed a mass in the right atrium (RA) and right ventricle (RV). The patient was subsequently started on antibiotics for pneumonia and was admitted to the medical intensive care unit. Further imaging was obtained. CT scans with and without contrast (Figure 1) showed a large filling defect involving the RV, measuring 7.3 x 3.7 cm; it was contiguous with a heterogeneous filling defect in the inferior vena cava (IVC). There was also a filling defect in the right hepatic vein and a large multiseptated heterogeneous right lobe liver mass measuring 11.5 x 8.6 x 12.2 cm.

**Figure 2:**
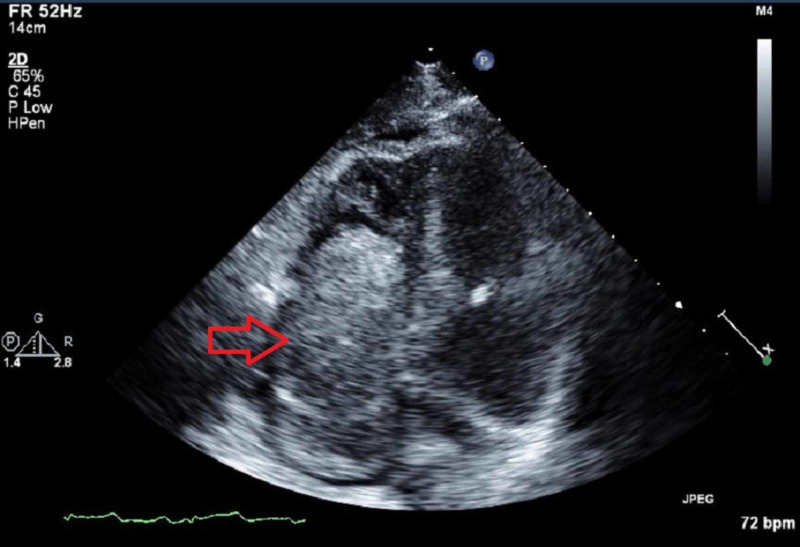
Apical four-chamber transthoracic echocardiogram view showing the mass in the right side of the heart.

**Figure 3:**
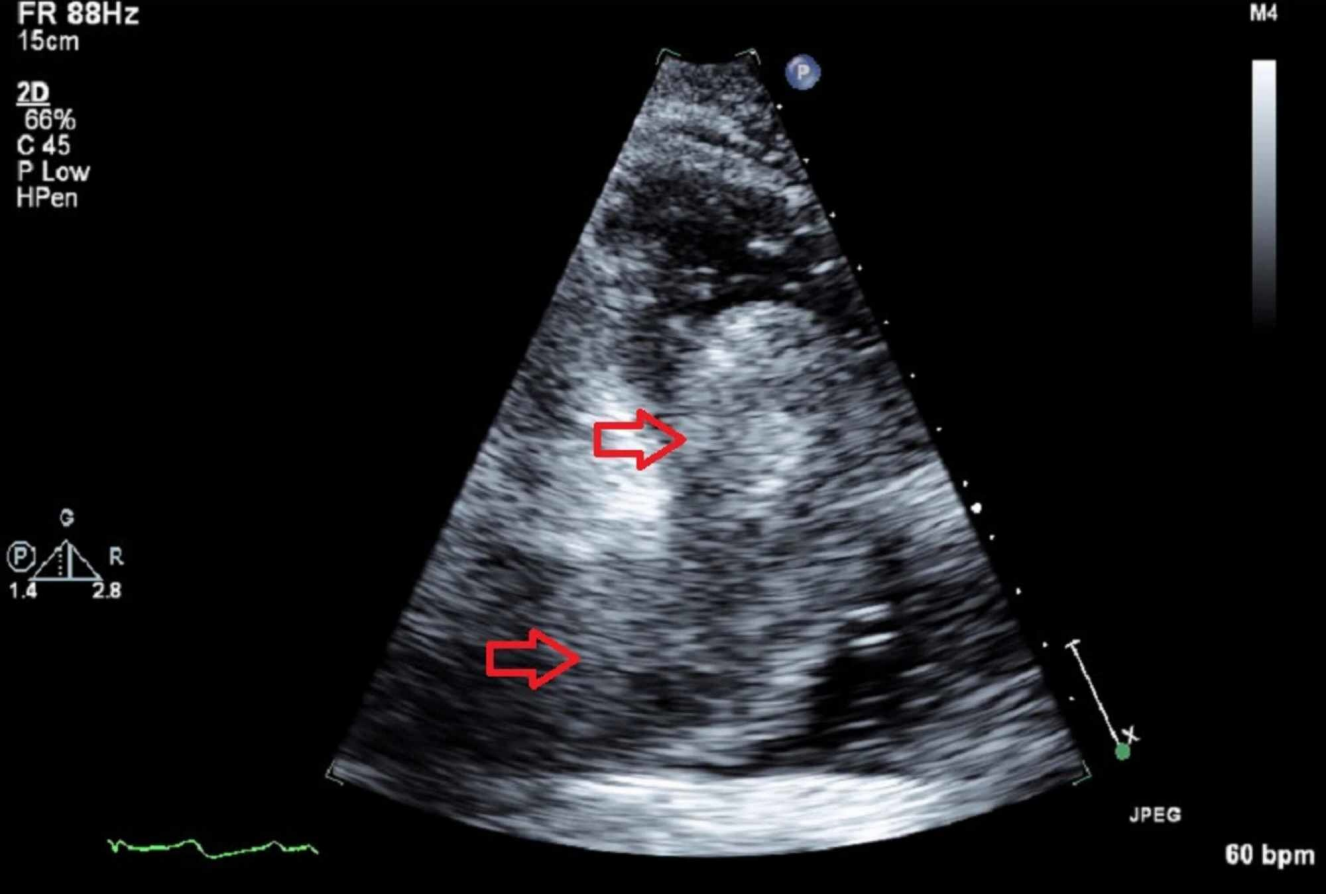
Apical two-chamber transthoracic echocardiogram view shows the mass in the right side of the heart. The arrow at the bottom shows the mass when entering the right atrium.

**Figure 4:**
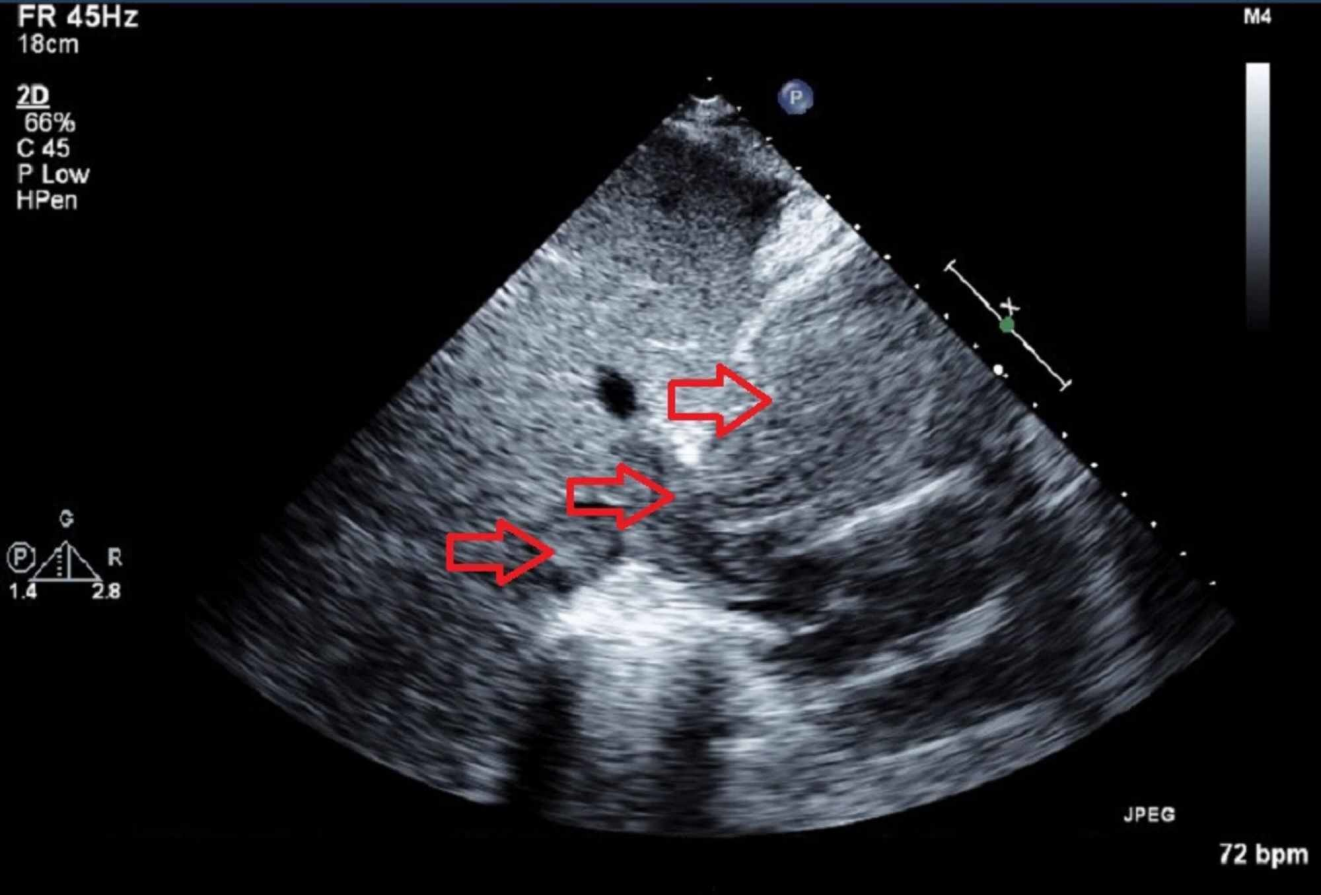
Subcostal transthoracic echocardiogram view shows the mass in the inferior vena cava and hepatic veins.

Transthoracic echocardiogram (TTE) (Figure 2-4) showed a large echodensity measuring 8 x 4 cm entering the RV from the RA; the mass appeared to originate from the liver, hepatic veins, and IVC and extend to the RA and RV, partially obstructing the RVOT. CT scans were also suggestive of cirrhosis. Alpha-fetoprotein (AFP) was 14.8 IU/mL, which is highly suggestive of HCC. Triple-phase chest abdomen CT (with a venous phase) helped to determine that the masses in the liver and heart were contiguous and of the same origin. 

Management was challenging, and a multidisciplinary team was involved. During the hospital stay, intravenous fluids were utilized to attempt keeping the RVOT open. Given history, presentation, imaging and AFP levels, oncology thought that the mass was HCC and they determined the prognosis to be grim. Cardiothoracic surgery advised that patient was not a surgical candidate. Comfort care measures were pursued, and the patient expired after five days of presentation.

## Discussion

This case demonstrates a rare cause of cardiac dysfunction, a cardiac tumor. Secondary cardiac tumors are more common than primary cardiac tumors [2-4]. Malignant melanomas are likely to metastasize to the heart [6-9]. Other solid tumors that can be associated with cardiac involvement are HCC, lung cancer, breast cancer, soft tissue sarcomas, renal carcinoma, esophageal cancer, and thyroid cancer [10]. Secondary cardiac tumors can occur secondary to hematogenous metastases, direct invasion from the mediastinum, or tumor growth into the vena cava and extension into the RA [11]. Presentation depends on location, and can include evidence of embolization, myocardial impairment, surrounding tissue invasion, valvular abnormalities, obstructive shock, or constitutional symptoms [12-14]. In our case, HCC has grown into the IVC and extended to the right side of the heart causing RVOT obstruction. Limited cases have been reported in the literature, and the management in those cases has been mostly surgical [15]. There has been a report about using transcoronary chemoembolization as well [16]. The survival rate was less than one year in most cases. Prognosis was thought to be grim in our case, and the patient expired [5,15,17]. Imaging in cardiac tumors is of extreme importance. Point-of-care echocardiogram is an important, fast to perform modality in shock status and can help in shock differentiation. TTE is the simplest, least invasive imaging modality to be utilized in cardiac tumors. Triple-phase CT has a diagnostic utility of better identifying and characterizing masses present in the venous system. AFP was utilized in this case, as suggested by studies, which showed that an AFP cut-off of 10.9 IU/mL would result in a sensitivity of 66% [18]. AFP when low or moderate does not rule out the diagnosis. In fact a large database of 1,773 HCC patients in Turkey was examined, in which 41.9% had AFP levels <20 IU/mL [19].

## Conclusions

Management of cardiac tumors is rather challenging, and is still need to be better studied and established. Secondary cardiac tumors are more common. Presentation can include circulatory shock. Point-of-care echocardiogram is a relatively easy, fast to perform test that helps in differentiating shock. Moreover, when used in the right context, AFP can help diagnosing HCC.
